# Membranous Rhinitis

**Published:** 1889-04

**Authors:** 


### *MEMBRANOUS RHINITIS.*

ABSTRACT.

Dr. Frank H. Potter, in the *Journal of Laryngology* for March, 1889, calls attention to certain forms of rhinitis, which he describes as membranous, on account of the principal symptom associated with them. This was the formation of a membrane over the inflamed surfaces. It appeared about the third day of the disease, and resembled that seen in diphtheria in some particulars. It was dead white and opaque; it was firmly attached to the parts beneath; it was only detached with violence, and left a bleeding surface. No trace, however, of diphtheria was found in these cases. The constitutional disturbances were but slight and easily controlled. The disease seemed to be local, and was especially annoying from its intensity and deviation. The author calls attention, also, to the fact that cocaine had very little influence over the swollen tissues. He used it in solutions from four to twenty per cent., and also combined with antipyrine, as suggested by Dr. Hinkel, of Buffalo, but in neither case did it exhibit its contractile power. The best results were obtained by packing carefully the nasal passages with pellets of dry absorbent cotton, allowing them to remain a short time, and then removing them and introducing others. In this way, the edema of the tissues was partially overcome, and gave relief for a short time. Along with these local measures, constitutional remedies were also given as atropia, salicylate of soda, bromide of potash, etc., but, notwithstanding, the disease appeared to run its course uninfluenced by treatment. After lasting about three weeks, the symptoms began to subside, and in a short time the disease had disappeared. During all this time the patients attended to their usual occupations, and complained only of the local distress.

There is very little literature on the subject, although membranous inflammation of the nasal passages is occasionally noticed. Dr. William A. Hammond, in the *Virginia Medical Monthly* for November, 1887, reports an attack in himself, and describes it in detail. In his case, as in those reported by Dr. Potter, there was no tendency in the disease to spread beyond the nose; even the pharynx exhibited but slight evidence, if any, of disturbance.

In conclusion, the author says: "The drift of opinion to-day is towards a broader conception of the nature of disease. It is expressed, in this instance, by most observers holding that all inflammation of the air passages associated with the formation of a false membrane are related, and this relation is indicated by saying they are expressions of the diphtheritic poison. Ought not this opinion to be modified by saying that the great *majority* of these cases are so related? Is the rule quite so universal as is usually held? If we can have an inflammation of the nose characterized by the formation of a false membrane, in which no trace of the diphtheritic poison can be discovered, cannot the same kind of an inflammation appear in the larynx or the pharynx? Can it not also occur in the bronchial tubes, and produce there a croupous bronchitis?"

These cases are reported from their clinical side, and are intended to be suggestive merely in regard to the questions involved in their consideration.

